# Change in Phylogenetic Community Structure during Succession of Traditionally Managed Tropical Rainforest in Southwest China

**DOI:** 10.1371/journal.pone.0071464

**Published:** 2013-07-31

**Authors:** Xiao-Xue Mo, Ling-Ling Shi, Yong-Jiang Zhang, Hua Zhu, J. W. Ferry Slik

**Affiliations:** 1 Center for Integrative Conservation, Xishuangbanna Tropical Botanical Garden, Chinese Academy of Sciences, Mengla, Yunnan, China; 2 Graduate University of Chinese Academy of Sciences, Beijing, China; 3 Department of Organismic and Evolutionary Biology, Harvard University, Cambridge, Massachusetts, United States of America; Michigan State University, United States of America

## Abstract

Tropical rainforests in Southeast Asia are facing increasing and ever more intense human disturbance that often negatively affects biodiversity. The aim of this study was to determine how tree species phylogenetic diversity is affected by traditional forest management types and to understand the change in community phylogenetic structure during succession. Four types of forests with different management histories were selected for this purpose: old growth forests, understorey planted old growth forests, old secondary forests (∼200-years after slash and burn), and young secondary forests (15–50-years after slash and burn). We found that tree phylogenetic community structure changed from clustering to over-dispersion from early to late successional forests and finally became random in old-growth forest. We also found that the phylogenetic structure of the tree overstorey and understorey responded differentially to change in environmental conditions during succession. In addition, we show that slash and burn agriculture (swidden cultivation) can increase landscape level plant community evolutionary information content.

## Introduction

Phylogenetic community structure analysis has proven to be an important new tool for differentiating the respective roles of environmental filtering and species interactions in shaping plant community structure [1]–[5]. Although exceptions do occur, most species traits are to some extent phylogenetically conserved, meaning that closely related species usually share similar traits and habitat preferences [3], [6]–[8]. If the environment is the main driver of community assembly, then species will be filtered for specific traits that enhance survival, growth and reproduction in specific habitats, leading to co-occurrence of closely related species with similar traits (phylogenetic clustering). If, however, species interactions (*e.g.* competition) drive the community assembly, then species with similar traits will start out-competing each other, leading to phylogenetically random or overdispersed species communities. Here we apply this principle to assess the impact of human disturbance on species community assembly along a successional gradient in a traditionally managed forest landscape.

Most of the tropics are now dominated by secondary forests [9], and these forests are therefore increasingly important for biodiversity conservation purposes [10]. Although many studies exist that describe different regeneration pathways of tropical secondary forests [11] very few try to identify the underlying mechanisms driving the community assembly process during succession. The few available studies suggest that phylogenetic clustering dominates during the early stage of succession, indicating that environmental filtering is shaping the community assembly during this phase, while later stages of succession generally show phylogenetic randomization or overdispersion suggesting that competitive interactions are dominating community assembly during that phase [12]–[14]. However, different types of human forest use and natural disturbances may influence successional pathways differentially, which will be reflected in the phylogenetic structure of a community [3], [8], [15]. Given the paucity of data currently available, it is important that phylogenetic community structure analysis is applied to as wide a range of disturbance types and successional pathways as possible so that the generality of the results so far can be assessed.

Slash and burn (swidden) cultivation is a typical traditional forest use type in Southeast Asia [16]. Traditional slash and burn cultivation on a limited scale increases rather than decreases landscape level tree diversity [17], although this outcome strongly depends on the distance between forest types for successful exchange of propagules [18]. In addition, secondary forests after slash and burn can harbor many endangered and red-list species that are rare or absent in primary forests [17]. However, although this earlier study [17] described diversity and composition patterns along the disturbance gradient, it did not explore the community assembly mechanisms driving the observed patterns.

Here we use phylogenetic community structure analysis to determine which process (environmental filtering versus species interactions) is driving species co-existence during succession after slash and burn agriculture. The objectives of our study were (1) to understand the successional pathways after slash and burn agriculture in terms of abiotic (environmental) and biotic (species interactions) drivers, and (2) to determine the impact of traditional forest use on phylogenetic diversity and community phylogenetic structure.

## Materials and Methods

### Study Site and Plot Setting

**Figure 1:**
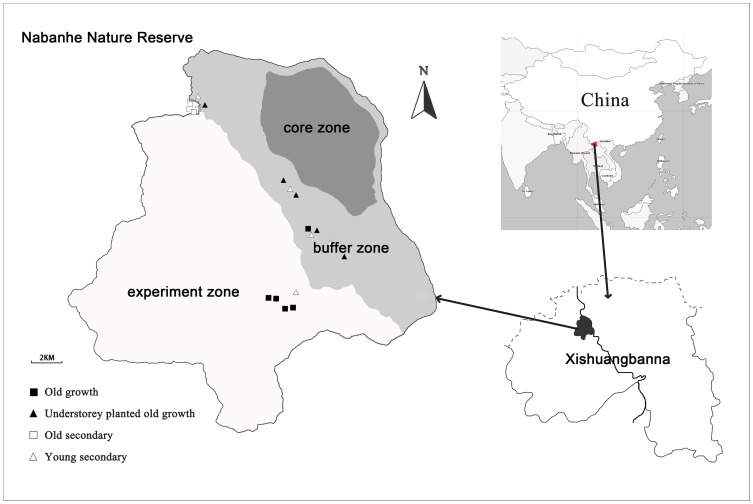
Map of the Nabanhe National Nature Reserve. Locations of the 20 plots are indicated as follows: Open squares indicate old secondary forest, closed squares represent old-growth forest, open triangles represent young secondary forest, and closed triangles represent forest with *Amomum villosum* plantation in the understorey.

The study was located in a tropical seasonal rainforest in Nabanhe National Nature Reserve (NNNR), Xishuangbanna, Yunnan province, China (22° 04′–22° 17′ N; 100° 32′–100° 44′ E). Permission to study in the reserve was given by the Nabanhe National Nature Reserve Bureau, which is located within the reserve. We studied four types of forest (five 500 m^2^ plots each) with different disturbance (management) histories: (i) old-growth forest that was open to understorey non-timber product collection, (ii) old-growth forests with understorey *Amomum* (Zingiberaceae) plantation, (iii) old secondary forests about 200-years after slash and burn, and (iv) young secondary forest about 15 to 50-years after slash and burn (Fig. 1).

Slash and burn is a typical land use/forest management type in tropical China and surrounding countries such as Myanmar, northern Laos, northern Vietnam and northern Thailand [16]. In this system, old-growth forests are burned for agricultural use and then abandoned after 7 to 8 years, after which natural succession takes over. Young secondary forests, as used in the present study had been regenerating for 15 to 50 years after abandonment while the old secondary forest had been abandoned about 200 years ago. Ages of the plots were determined by questioning local people from villages near each plot and by looking at the size of long lived pioneer species known to establish after slash and burn, such as *Duabanga grandiflora* (Lythraceae). Slash and burn was not repeated after abandonment. Slash and burn based agriculture usually only provides a food supply for the farmers family and very little extra income can be generated. The understorey planting of ginger *Amomum villosum*, a Chinese medicinal plant, was promoted by the government in the 1970s to improve the income of local people, and was then favored over slash and burn because of the relatively high income generated.

### Field Survey and Plant Inventory

In the 20 plots (25×20 m, except for the old secondary forest where plots were 10×50 m because this forest was located in a narrow valley), all trees with a diameter at breast height (dbh) ≥5 cm, and all lianas were identified and their diameter measured. Most previous analyses in Xishuangbanna used the 20×25 m plot shape [19], [20]. Following the plot design in this area allows further analysis combining our data with the data from other forest areas in this region. Longer plots (10×50 m) used in narrow valleys may capture more beta-diversity and landscape variability, so they might overestimate diversity. However, this overestimation is probably limited because of the small plot size. Treelets (with a dbh <5 cm), seedlings, shrub and herb species were identified in five 2.5×2 m subplots (four corners and the center) within each 500 m^2^ plot. Vouchers were collected for plant species that could not be identified in the field and were deposited in the Herbarium of Xishuangbanna Botanical Garden (HITBC), CAS, Menglun, Yunnan, China.

### Wood Density

Wood density, a good indicator of the successional stage and conservation value of tropical forest in Asia [21], was taken from a global data set compiled from previously published values [22], [23]. When the wood density of the tree species was not available in the data set, the average of the genus that the species belonged to was used. This approach is reliable because wood density is a strongly phylogenetic conservative trait in tropical trees [24]. The wood density of each plot was weighted by the basal area of each species.

### Construction of the Community Phylogenetic Tree

We constructed a phylogenetic tree (File S1, S2) with the APGIII classification as a backbone for all the species in our study site using the program PHYLOMATIC v 4.0.1 [25]. However, this tree was only resolved up to family level. To increase the resolution of this phylogeny we recovered rbcL (548–697 bp) sequences for 163 species from GenBank. For another 76 species in our dataset, for which no rbcL data was available, we assigned the rbcL sequence of the closest available congener. This is a reasonable approach since rbcL is a slowly evolving sequence and shows very little variation below the genus level [26], [27]. Five species for which we found no rbcL sequences and which also had no congeneric relative with known rbcL sequence were excluded from the analysis. These five species were rare in the sampled communities, and should not bias our further analyses, especially as these individuals were not clustered into a particular phylogenetic lineage. Using the rbcL sequences, one most likely phylogenetic tree was built under the GTR model of evolution with a maximum likelihood approach using the software PAUP version 4 [28] with 100 bootstrap replicates to assess node support. The phylogenetic hypothesis built with the rbcL gene was broadly consistent with the topology of the APG III phylogeny. In the rbcL tree, most families had a bootstrap node support >80. Below the family level, most of the genera appeared well resolved although with lower bootstrap support values. We then used the APGIII phylogeny as generated by PHYLOMATIC as our family level backbone phylogeny and resolved the within family phylogenetic classification by adding the results obtained from the rbcL phylogeny. Branch lengths and dated nodes for this megatree were obtained by applying the *bladj* algorithm of PHYLOCOM [25], with calibration ages from [29].

### Phylogenetic Diversity and Community Structure

In order to standardize plot comparisons all diversity analyses described below were based on a fixed number (based on the plot with the fewest stems) of randomly selected individuals from each plot. This rarefaction procedure reduces the impact of sample size differences between plots on the diversity analyses [30]. We calculated phylogenetic species variability (PSV), a measure of the phylogenetic relatedness of species within a community, of each plot based on species presence/absence and their phylogenetic relationships within the rarefied samples [31]. Faith’s phylogenetic diversity (PD) [32] of each plot was calculated as the sum of branch lengths of the subtending tree of the species present in the rarefied samples. We calculated phylogenetic species richness (PSR) for each plot by multiplying the number of species in the rarefied samples by their PSV value, and calculated phylogenetic species evenness (PSE) by incorporating relative species abundances within the rarefied samples into PSV [31]. All these phylogenetic diversity indices were calculated by using PHYLOCOM [25] and the R package picante [33].

The phylogenetic similarity of the plant communities was assessed using Unifrac [34]. UniFrac estimates the distance between communities as the fraction of the branch length of the phylogenetic tree that leads to descendants from either one environment or another, but not both. We used the resulting distance matrix to cluster environments using Jackknifed UPGMAwith R package picante [33].

We calculated the abundance weighted net relatedness index (NRI) and nearest taxon index (NTI) of each plot to measure the phylogenetic dispersion (relatedness) of the co-occurring species by using PHYLOCOM [35]. The NRI and NTI for each rarified sample were calculated as:
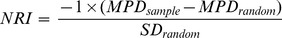
(1)

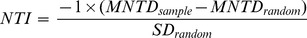
(2)


Where MPD is the mean pairwise phylogenetic distance between all individuals in each rarified sample and MNTD is the mean phylogenetic distance for each individual to its nearest relative within each rarified sample. The MPD_random_ and MNTD_random_ are the mean MPD and mean MNTD from 999 randomly generated assemblages. An independent swap null model was used to generate these 999 random assemblages. For both NRI and NTI, values close to zero indicate random phylogenetic structures, positive values indicate clustered, while negative values indicate overdispersed community phylogenetic structures.

### Data Analysis

Species-individual curves show the relationship between number of species and number of individuals and were generated with the program EstimateS (v. 7.5) [36]. To generate species-individual curves for each of the 20 plots, individuals recorded in each plot were randomly sorted and the species number accumulation was tallied 50 times to get the mean value. This analysis was repeated for all individuals within each forest type to construct the species-individual curves of the four studied forest management types. In addition, we combined all individuals from the 20 plots to generate a landscape level species-individual curve.

PD-individual curves show the relationship between phylogenetic diversity and number of individuals. We obtained PD-individuals curves for each plot, for each of the four forest types, and for all the plots together. The curve was computed using R package “phylocurve” [37]. We also constructed phylogenetic species richness (PSR) accumulation curves using the function specaccum.psr in the “picante” package with the method rarefaction [3], [33].

One-way ANOVA analysis with a post-hoc Least Significant Difference (LSD) test was used to test whether rarified tree species number per plot, PD, PSV, PSR, average wood density and liana abundance were different among the four forest types. When, even after data transformation, unequal variances were detected between the four forest types for a test variable (which violates the basic assumptions for running an ANOVA), a non-parametric Kruskal-Wallis test was used instead.

## Results

**Figure 2:**
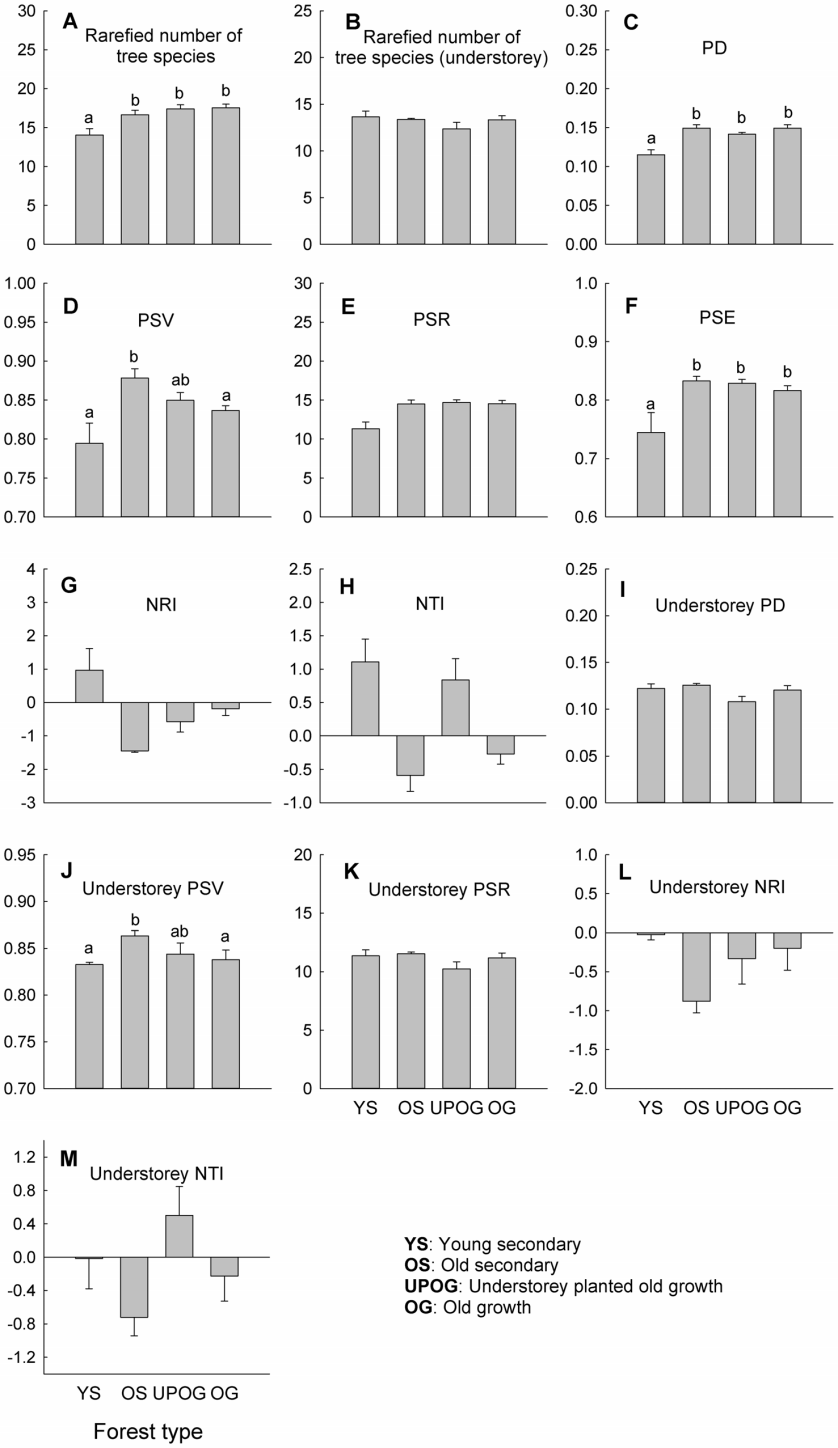
Species and phylogenetic diversity, and phylogenetic structure of overstorey and understorey trees for different types of forest. All the values are based on rarefied values. PD = phylogenetic diversity; PSV = phylogenetic species variability; PSR = phylogenetic species richness; PSE = phylogenetic species evenness; NRI = net relatedness index; NTI = nearest taxon index. Bars are means+standard error (SE). Bars topped by different letters indicate significant differences between forest types (P<0.05).

Rarefied number of tree species, phylogenetic diversity (PD) and phylogenetic species evenness (PSE) were significantly lower in the young secondary forest than in the other forest types (Fig. 2). However, understorey tree species number per plot did not differ between forest types (Fig. 2). Phylogenetic species variability (PSV) was highest in the old secondary forest (Fig. 2), but no significant differences were found in phylogenetic species richness (PSR) among different types of forest, even though the PSR of the young secondary forest was lower than that of other forest types (Fig. 2). The net relatedness index (NRI) of the young secondary forest was positive, while that of the other three types of forest was negative (Fig. 2). The nearest taxon index (NTI) of the young secondary forests and understorey planted forests were positive, while that of the other two types were negative (Fig. 2). Understorey PD and PSR were similar between forest types (Fig. 2), while understory PSV was highest in old secondary forest (Fig. 2). Understorey NRI of the young secondary forest was close to zero, while that of the other forest types was negative (Fig. 2). Understorey NTI of the young secondary forests was close to zero, while that of understorey planted forests was positive, and that of the old secondary and old growth forests were negative (Fig. 2).

**Figure 3:**
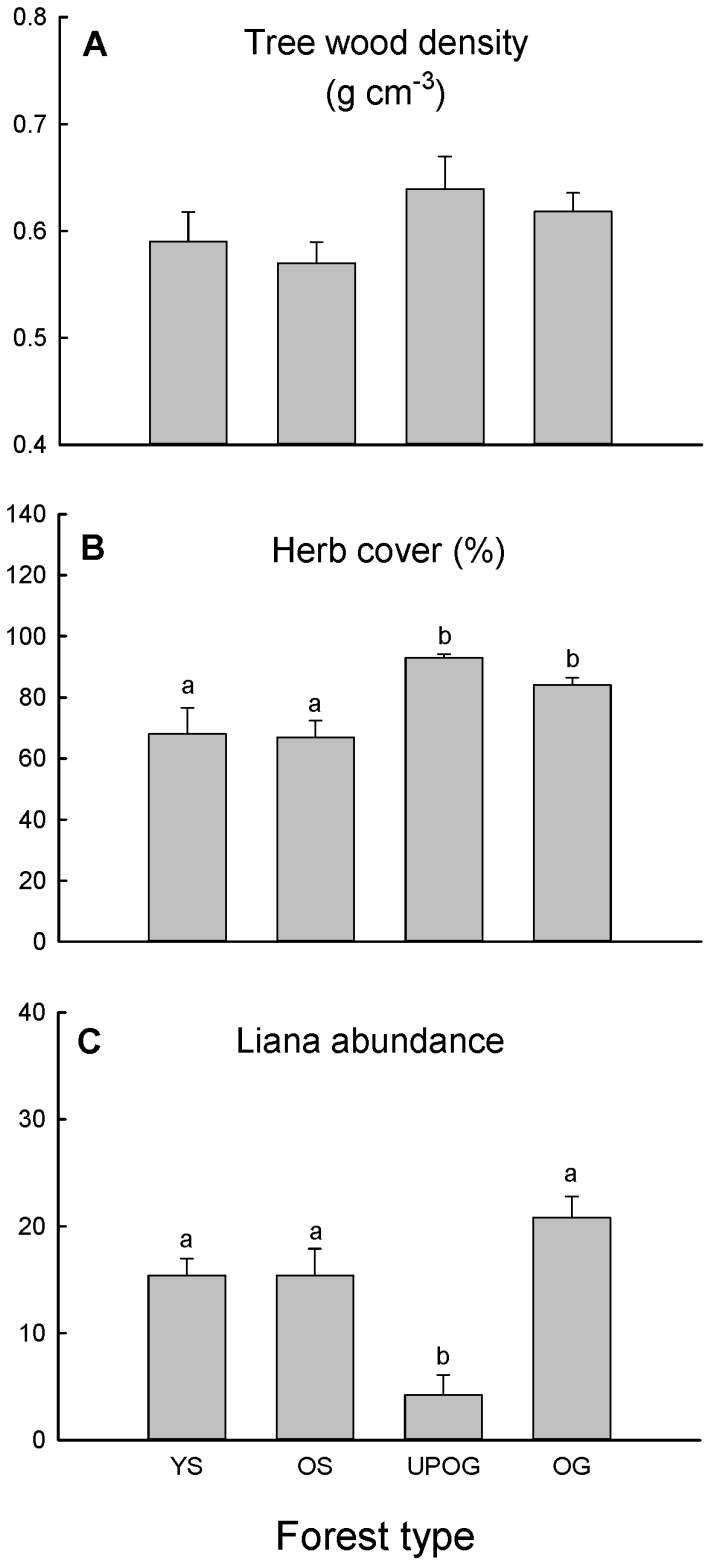
Basal area weighted tree oven-dry wood density, percent herb cover, and liana abundance for different forest types. YS represents young secondary forest; OS represents old secondary forest; UPOG represents understorey planted old-growth forest (the old-growth forests with *Amomum villosum* plantation in the understorey); OG represents old-growth forest. Bars are means+standard error (SE). Bars topped by different letters indicate significant differences between forest types (P<0.05).

No significant difference was found in basal area weighted average wood density among different types of forest (Fig. 3), but percent herb cover was significantly lower in young and old secondary forests than in old growth and understorey planted forests (Fig. 3). Liana abundance was lowest in the understorey planted forests (Fig. 3).

**Figure 4:**
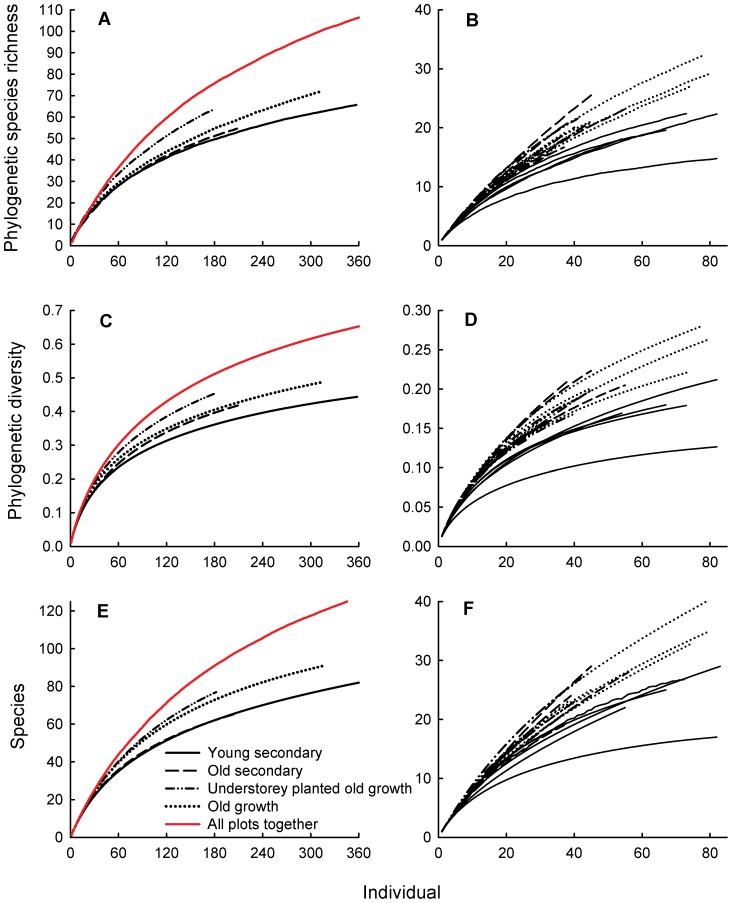
Phylogenetic species richness (PSR), diversity (PD) and species-individual curves: (A; C; E) curves for each forest type and all forest types combined, and (B; D, F) curves for the 20 individual plots.

**Figure 5:**
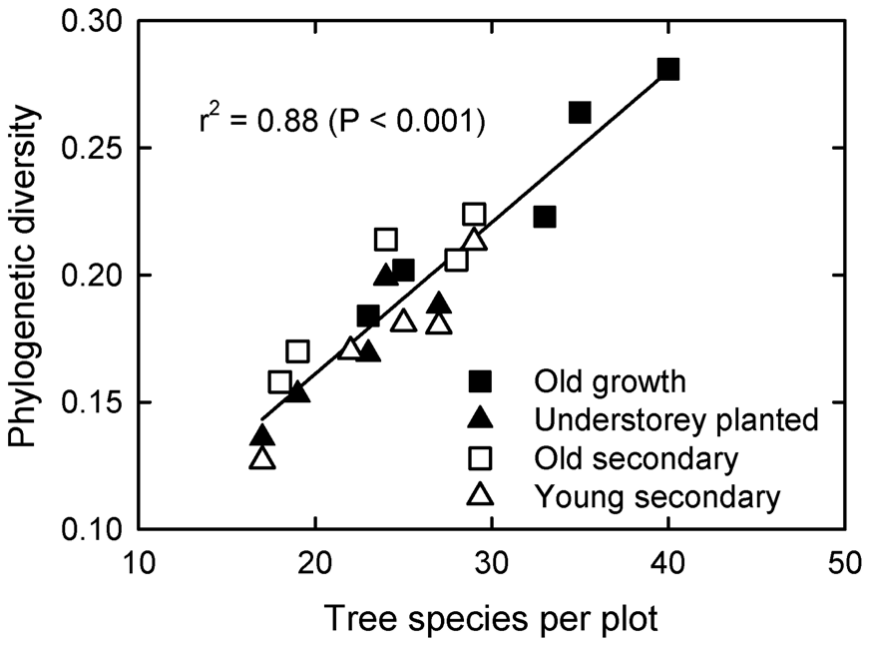
The relationship between phylogenetic diversity and tree species per plot. Open squares indicate old secondary forest, closed squares represent old growth forest, open triangles represent young secondary forest, and closed triangles represent forest with *Amomum villosum* plantation in the understorey.

The PD and PSR accumulation curves of the forest types showed a similar pattern as the species accumulation curves (Fig. 4). The young secondary forest plots showed markedly lower PD, PSR and species accumulation than other types of forest when the individual plots were analyzed (Fig. 4). However, when all plots within a forest type were combined, different types of forests showed similar PD, PSR and species accumulation (Fig. 4). Additionally, when all plots from different forest types were combined, the accumulation of PS, PSR and species number were faster and eventually higher than that of all forest types separately (Fig. 4). Species accumulation of the old secondary forests was completely overlapping with young secondary forests, while PD accumulation of the old secondary forests was almost completely overlapping with the old-growth forests (Fig. 4). Phylogenetic diversity was positively correlated with number of tree species per plot (Fig. 5), but not with PSV (relationship not shown).

**Figure 6:**
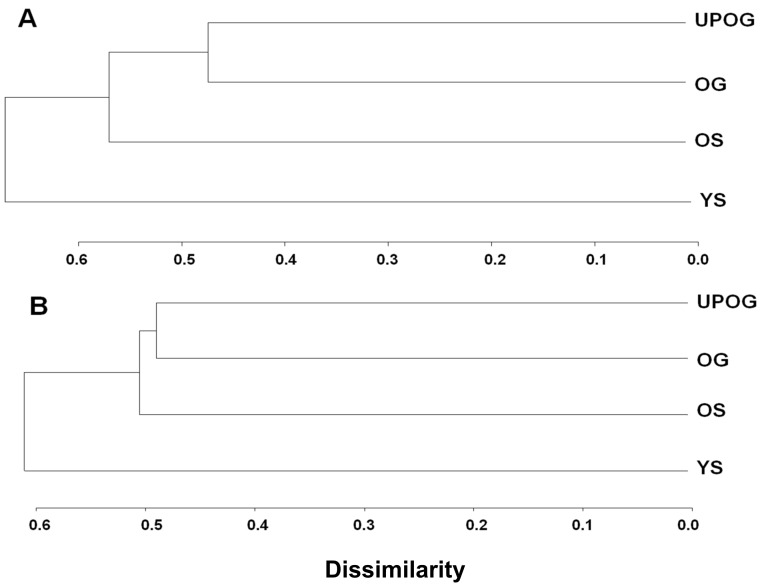
Phylogenetic dissimilarity of the plant communities based on overstorey (A) and understory (B) data from all plots per forest type (UPGMA clustering of the Unifrac distance matrix). YS represents young secondary forest; OS represents old secondary forest; UPOG represents understorey planted old-growth forest (the old-growth forests with *Amomum villosum* plantation in the understorey); OG represents old-growth forest.

For the overstorey, the community phylogenetic similarity analysis (UniFrac) distinguished the young secondary forest from other forest types (UniFrac; *P*<0.05). In addition, the level of similarity between underplanted forest and old growth forest was higher than that between old growth forest and old secondary forest, and between underplanted forest and old secondary forest (Fig. 6). The cluster pattern of the understorey was similar to that of the overstorey (Fig. 6).

## Discussion

Our study reveals the change in phylogenetic community structure of the overstorey and understorey along a successional gradient for a seasonal tropical rainforest in China. By studying the recovery of species and phylogenetic diversity after slash and burn, a typical traditional forest management type in SE Asia, our study also provides insight into the maintenance of diversity in human inhabited rainforests.

### Change in Phylogenetic Community Structure with Succession

The phylogenetic structure (NRI) of the tropical rainforest in China shifted from clustering in young secondary forests to over-dispersion in old secondary forests, and then to random (no pattern) in old growth forests. This change in phylogenetic structure (NRI) from clustering to over-dispersion along the successional gradient of this tropical rainforest in China generally agrees with findings from the few available other studies that estimated phylogenetic community structure in woody plants along succession gradients [13], indicating that this pattern may be applicable across the tropics and across a range of disturbance types. The detected shift from phylogenetic clustering, to over-dispersion to randomization along the successional gradient suggests a shift from community assembly mainly governed by environmental filtering during the initial phase of regeneration towards community assembly governed by strong competitive interactions during later successional phases, and finally a community assembly governed by a balance between environmental filtering and competitive interactions in old growth forests.

Interestingly, NTI showed an opposite pattern with NRI for the underplanted old growth forests in both the understorey and overstorey, signaling that although this forest type is characterized by a phylogenetically overdispersed tree community when deep (old) phylogenetic nodes are considered, this same tree community is clustered when only shallow (recent) nodes are considered. This means that within families and/or genera, species were more closely related than expected by chance, while at order and family level the forest was phylogenetically overdispersed. This may represent an artificial effect caused by the manual thinning that was carried out in this forest type to enhance growth conditions for the underplanted *Amomum villosum* (a ginger). Thinning may result in disappearance of some of the rarer species from the community, which is more likely to affect NTI than NRI simply because disappearance of species will immediately affect phylogenetic patterns within shallow nodes, but is unlikely to lead to changes in the older nodes (it is less likely that a species disappearance leads to disappearance of a whole plant family or order from the community, but it does immediately affect the number of co-occurring species within genera).

Young secondary forests are generally dominated by species that are adapted to fast colonization of open areas (pioneers), characterized by small and widely dispersed seeds, fast growth, low shade tolerance, high photosynthetic rates and large leaves with low length/width-ratios that are continuously shed and renewed (short leaf life-spans) [38]. This limited set of often extremely successful and environmentally well adapted colonists which establish directly after disturbance leads to a plant community dominated by a few closely related plant taxa within a limited set of plant families, hence the strongly phylogenetically clustered nature of these early secondary vegetations [5].

In general, the life-spans of early successional species are short (∼30 years) and they usually fail to regenerate under their own canopy due to shade intolerance, leading to a gradual decline in abundance of early successional species during forest regeneration. Therefore, if seed limitation is no problem (i.e. late successional species can reach the regenerating forest stand), the early succesional species will be replaced by late successional species which establish in the shady forest understorey. Most late successional tropical tree species are adapted to shade during their early establishment phase, so the number of phylogenetic lineages that can coexist during this phase is large and spread over almost the entire phylogeny. Therefore a decline in phylogenetic clustering is to be expected during later succesional stages. However, the strong phylogenetic over-dispersion that we detected during the later successional stage suggests that species traits linked to resource acquisition are phylogenetically conserved, i.e., species that are phylogenetically closely related will compete more intensely with each other for resources than they do with more distantly related species, eventually leading to the phylogenetically over-dispersed tree community that we observed [13], [39].

Several old growth tropical forests have been reported to be phylogenetically over-dispersed [12]–[14], [40]. However, we have observed phylogenetic randomization (absence of over-dispersion) in the old growth rainforests in the present study. The random pattern may suggest a more balanced coexistence of closely and distantly related species, i.e. competition and environmental filtering working simultaneously, resulting in a mix of overlapping and dissimilar traits. The difference of patterns compared to other observations could be related to the frequency of natural and human disturbances which is historically high in the Asian tropics [11], [41].

### Change in Phylogenetic Structure of the Forest Understorey

Unlike overstorey trees, understorey tree seedlings showed a randomized phylogenetic pattern in young secondary forests (corresponding to findings from Costa Rica) [12] and over-dispersion in old growth forests. The difference may be caused by the different environmental conditions experienced by understorey versus overstorey plants, i.e. the understorey, even in young secondary forests, consists of a shaded, relatively stable habitat. Such relatively homogeneous conditions might lead to an increased role of species interactions in the form of competition for soil nutrients, water and light, producing less phylogenetically clustered plant communities because competition for these scarce and patchy resources will be most intense between close relatives which generally share similar environmental requirements. Phylogenetic structure patterns also differed between size classes in a Costa Rican rainforest with smaller trees being more phylogenetically distant [12]. These results therefore suggest that the importance of species interactions and environmental filtering changes not only in time during succession, but also along size classes within forest tree communities. In fact, these changes in phylogenetic structure along the size classes reflect the successional change over time because the smallest tree diameter classes represent the future tree composition of these forests.

### Impact of Forest Management on Phylogenetic Diversity

Previously we found that traditional forest management has limited impact on plant diversity, and slash and burn agriculture even increased landscape level diversity [17]. As the young secondary forests had clustered phylogenetic structure and low PSR, our expectation was that they would have a limited impact on the regional phylogenetic diversity despite their high species richness. However, the PD accumulation curves of the secondary forest types was not lower than that of the old growth forest at the forest type level, while the PSR of old secondary forest was even higher than that of the primary forest. As the common species in our secondary forests were different from those of the old growth forests, as shown by the cluster analysis and the landscape scale diversity accumulation curves, traditional forest management as practiced at our study site not only increased species but also phylogenetic diversity at the landscape level.

### Conclusion

In conclusion, we revealed a change in phylogenetic community structure in a seasonal rainforest in tropical China along a >200 year regeneration timescale that went from clustering to over-dispersion to randomization. We also found that the overstorey and understorey phylogenetic structure responded to change in environmental conditions with succession in different ways, i.e. less phylogenetic clustering in the understorey. In addition, we confirmed the beneficial effects of slash and burn (swidden) cultivation on landscape level phylogenetic variability and diversity.

## Supporting Information

File S1 **Community phylogenetic tree used in the study.**
(PDF)

File S2 **Nexus format of the phylogenetic tree used in the study.**
(DOCX)
