# Supplementary figures and images for: Change in Phylogenetic Community Structure during Succession of Traditionally Managed Tropical Rainforest in Southwest China
Source: PLoS One. 2013 Jul 31;8(7):e71464. doi: 10.1371/journal.pone.0071464 (PMC3729948; doi:10.1371/journal.pone.0071464)

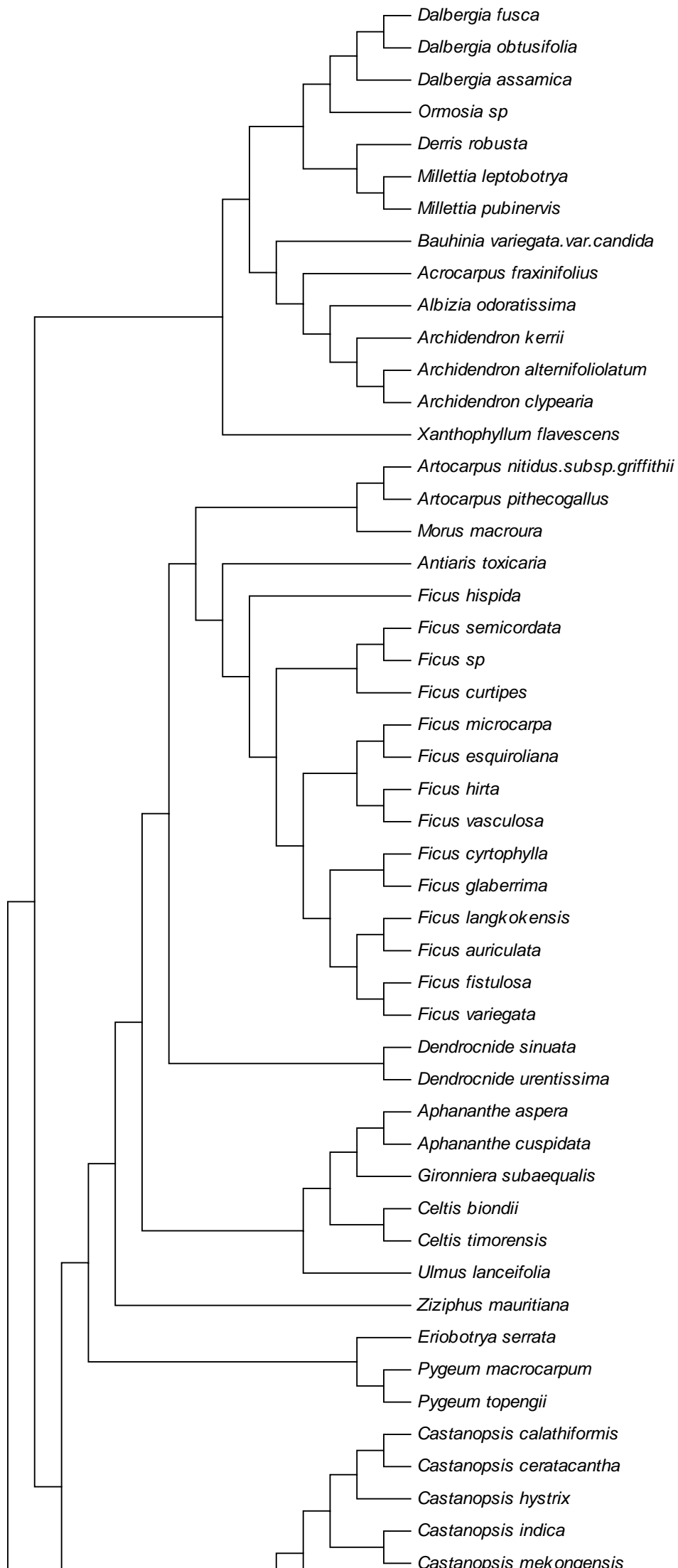

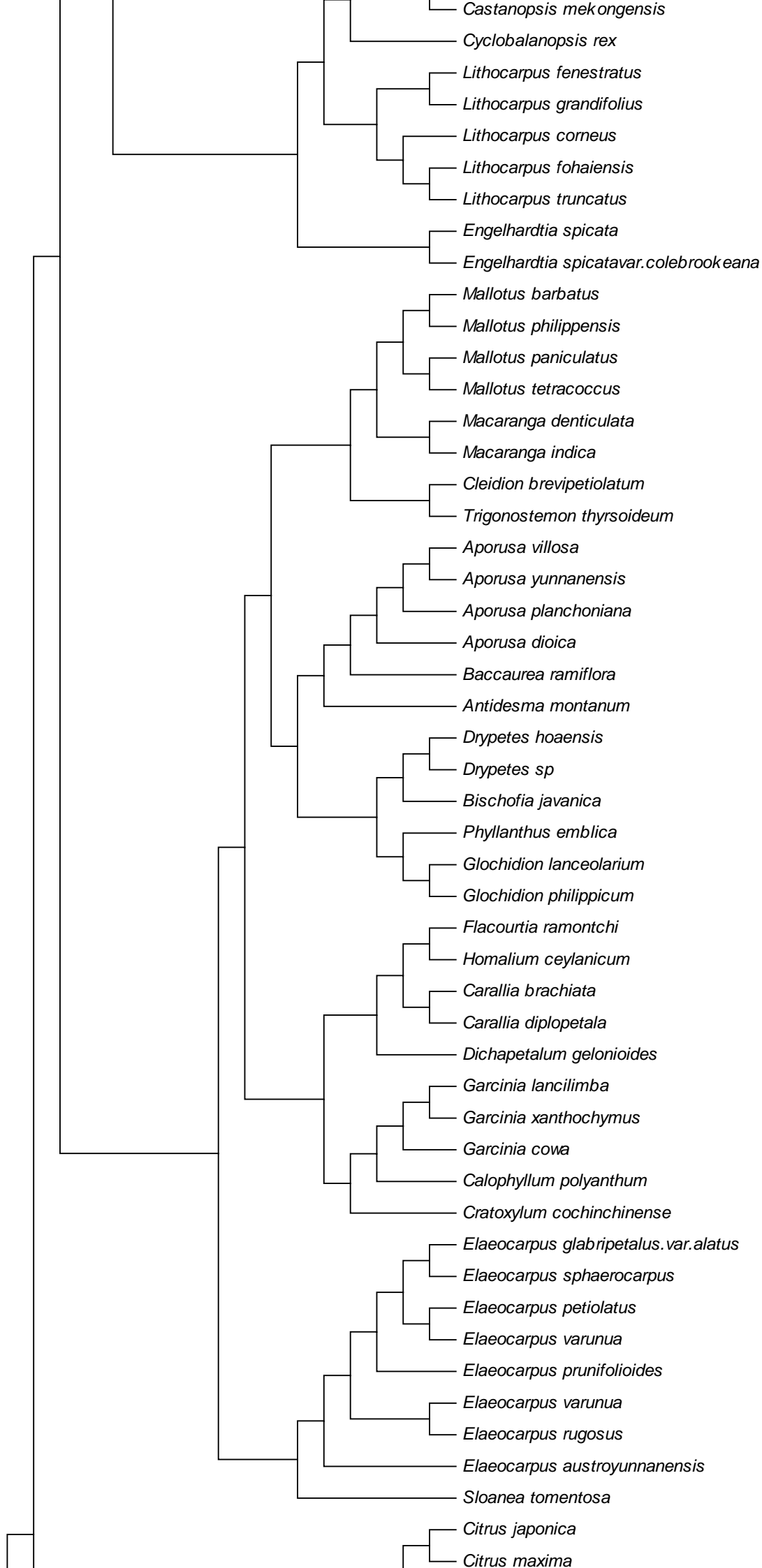

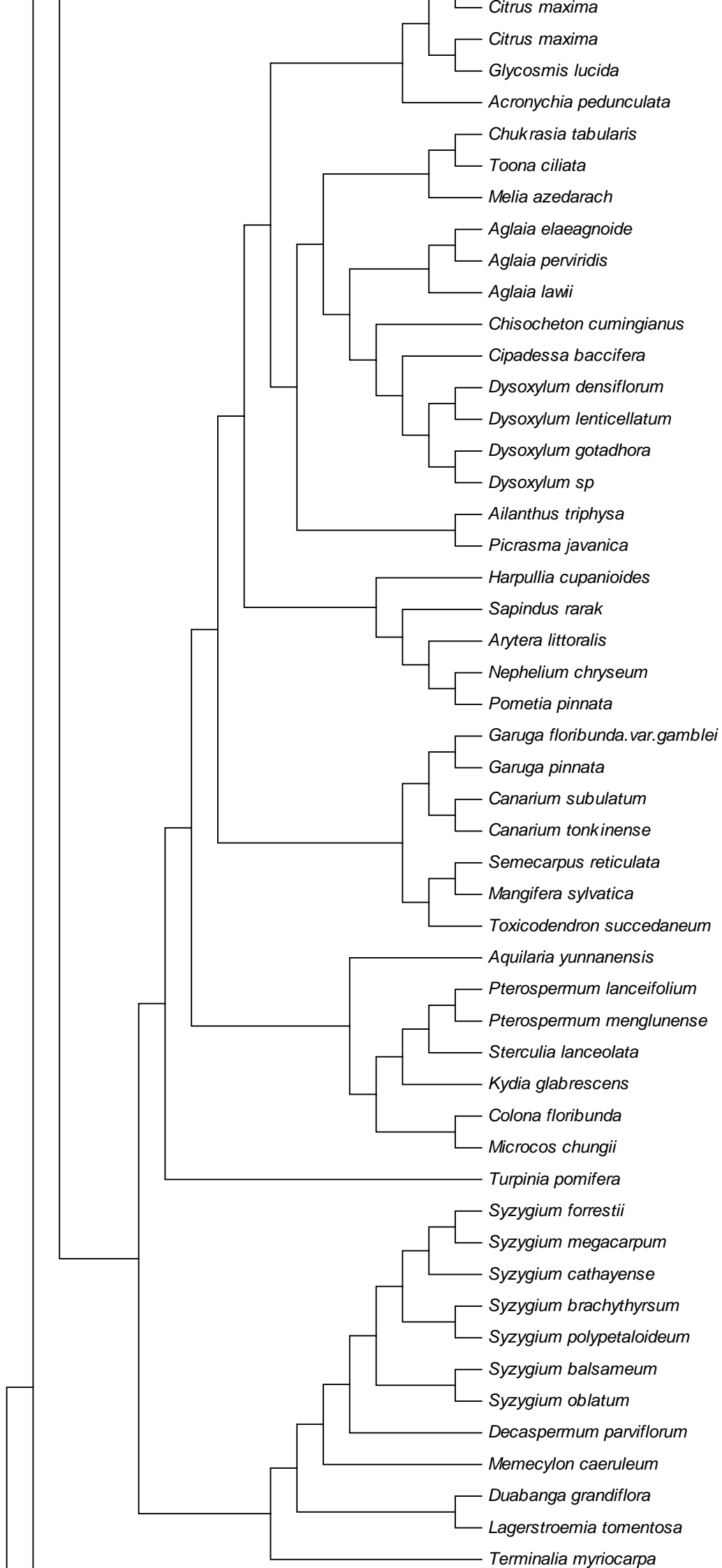

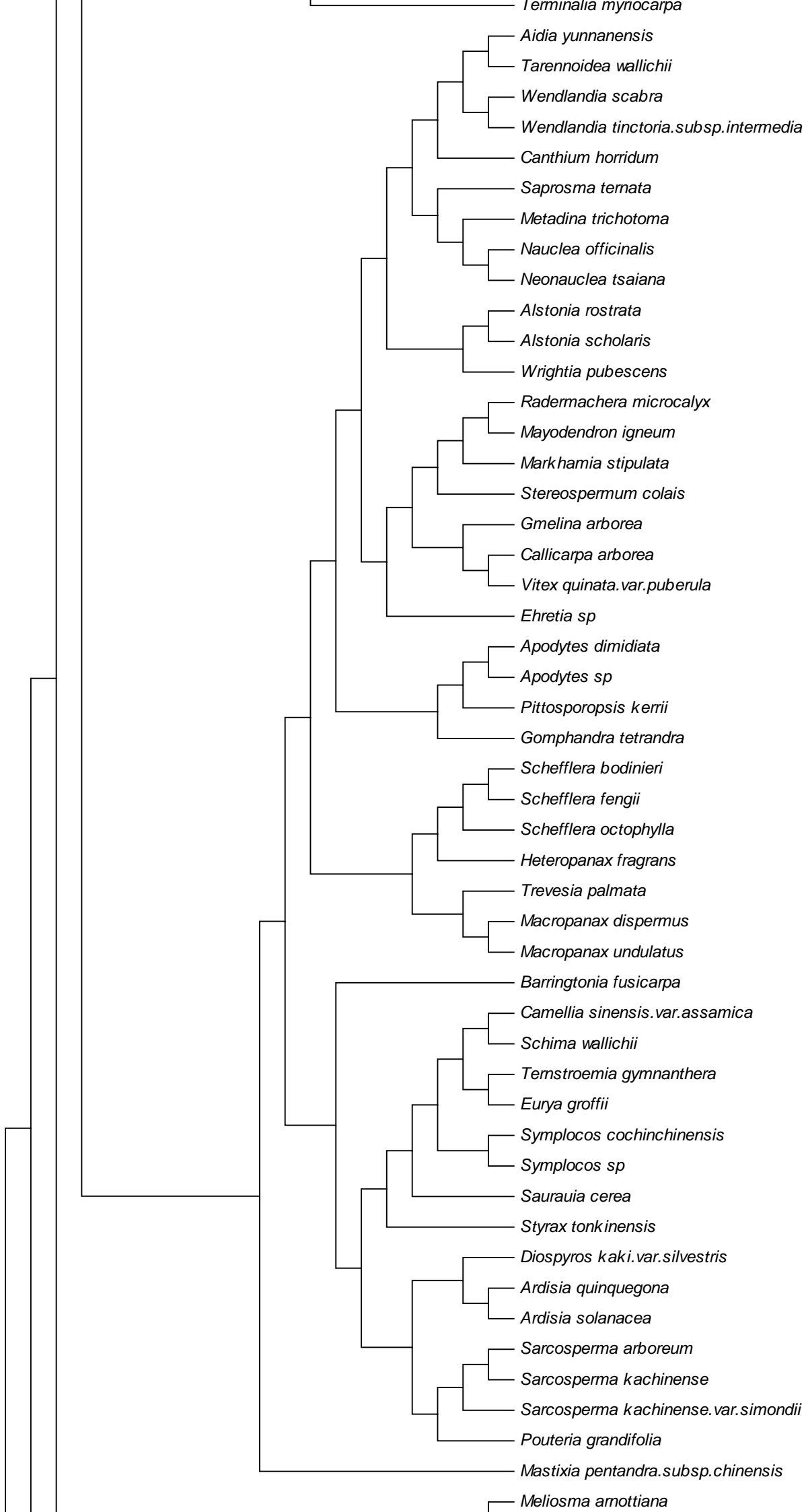

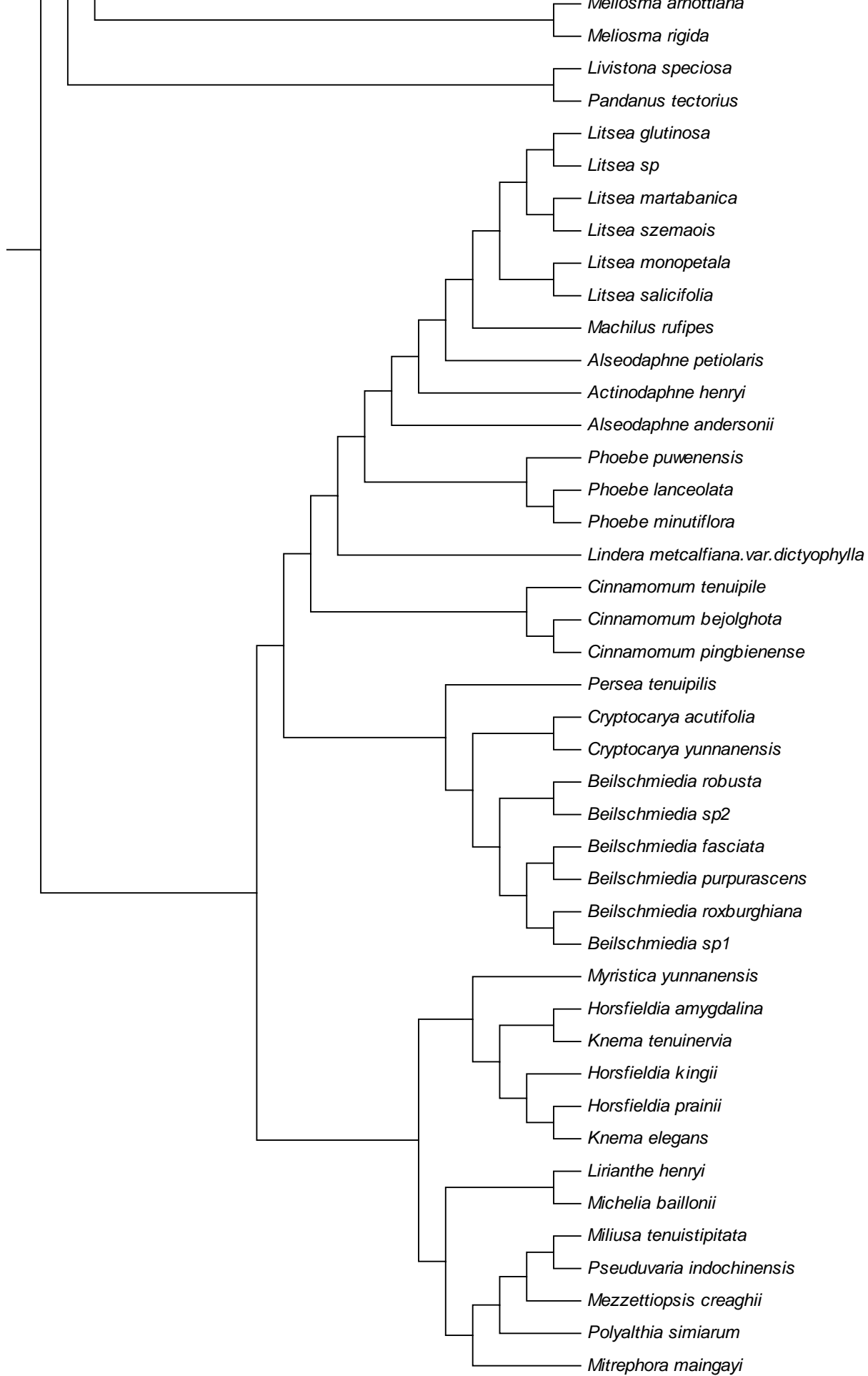

Supplement: File S1 — Community phylogenetic tree used in the study. (PDF) [file pone.0071464.s001.pdf]
