# Supplementary material for: Change in Phylogenetic Community Structure during Succession of Traditionally Managed Tropical Rainforest in Southwest China
Source: PLoS One. 2013 Jul 31;8(7):e71464. doi: 10.1371/journal.pone.0071464 (PMC3729948; doi:10.1371/journal.pone.0071464)
Supplement: File S2 — Nexus format of the phylogenetic tree used in the study. (DOCX) [file pone.0071464.s002.docx]

((((((((((((((dalbergia_fusca:40.000000,dalbergia_obtusifolia:40.000000):40.000000,dalbergia_assamica:80.000000):40.000000,ormosia_sp:120.000000):40.000000,(derris_robusta:106.666664,(millettia_leptobotrya:53.333332,millettia_pubinervis:53.333332):53.333332):53.333336):40.000000,(bauhinia_variegata.var.candida:166.666656,(acrocarpus_fraxinifolius:133.333328,(albizia_odoratissima:100.000000,(archidendron_kerrii:66.666664,(archidendron_alternifoliolatum:33.333332,archidendron_clypearia:33.333332):33.333332):33.333336):33.333328):33.333328):33.333344):33.333328,xanthophyllum_flavescens:233.333328):33.333328,((((((((artocarpus_nitidus.subsp.griffithii:50.793652,artocarpus_pithecogallus:50.793652):50.793652,morus_macroura:101.587303):50.793648,(antiaris_toxicaria:133.333328,(ficus_hispida:114.285713,(((ficus_semicordata:31.746033,ficus_sp:31.746033):31.746033,ficus_curtipes:63.492065):31.746033,(((ficus_microcarpa:25.396826,ficus_esquiroliana:25.396826):25.396826,(ficus_hirta:25.396826,ficus_vasculosa:25.396826):25.396826):25.396824,((ficus_cyrtophylla:28.571428,ficus_glaberrima:28.571428):28.571428,((ficus_langkokensis:19.047619,ficus_auriculata:19.047619):19.047619,(ficus_fistulosa:19.047619,ficus_variegata:19.047619):19.047619):19.047619):19.047619):19.047623):19.047615):19.047615):19.047623):19.047623,(dendrocnide_sinuata:85.714287,dendrocnide_urentissima:85.714287):85.714287):19.047623,((((aphananthe_aspera:38.095238,aphananthe_cuspidata:38.095238):38.095238,gironniera_subaequalis:76.190475):38.095245,(celtis_biondii:57.142860,celtis_timorensis:57.142860):57.142860):38.095230,ulmus_lanceifolia:152.380951):38.095245):19.047607,ziziphus_mauritiana:209.523804):19.047623,(eriobotrya_serrata:152.380951,(pygeum_macrocarpum:76.190475,pygeum_topengii:76.190475):76.190475):76.190475):19.047623,((((((castanopsis_calathiformis:35.374149,castanopsis_ceratacantha:35.374149):35.374149,castanopsis_hystrix:70.748299):35.374153,(castanopsis_indica:53.061226,castanopsis_mekongensis:53.061226):53.061226):35.374146,quercus_rex:141.496597):35.374146,((lithocarpus_fenestratus:66.326530,lithocarpus_grandifolius:66.326530):66.326530,(lithocarpus_corneus:88.435371,(lithocarpus_fohaiensis:44.217686,lithocarpus_truncatus:44.217686):44.217686):44.217690):44.217682):35.374161,(engelhardia_spicata:106.122452,engelhardia_spicata.var.colebrookeana:106.122452):106.122452):35.374146):19.047607):19.047638,(((((((mallotus_barbatus:40.000000,mallotus_philippensis:40.000000):40.000000,(mallotus_paniculatus:40.000000,mallotus_tetracoccus:40.000000):40.000000):40.000000,(macaranga_denticulata:60.000000,macaranga_indica:60.000000):60.000000):40.000000,(cleidion_brevipetiolatum:80.000000,trigonostemon_thyrsoideum:80.000000):80.000000):40.000000,((((((aporusa_villosa:28.571430,aporusa_yunnanensis:28.571430):28.571430,aporusa_planchoniana:57.142860):28.571426,aporusa_dioica:85.714287):28.571434,baccaurea_ramiflora:114.285721):28.571426,antidesma_montanum:142.857147):28.571426,(((drypetes_hoaensis:42.857143,drypetes_sp:42.857143):42.857143,bischofia_javanica:85.714287):42.857140,(phyllanthus_emblica:85.714287,(glochidion_lanceolarium:42.857143,glochidion_philippicum:42.857143):42.857143):42.857140):42.857147):28.571426):28.571442,((((flacourtia_ramontchi:47.619049,homalium_ceylanicum:47.619049):47.619049,(carallia_brachiata:47.619049,carallia_diplopetala:47.619049):47.619049):47.619049,dichapetalum_gelonioides:142.857147):47.619049,((((garcinia_lancilimba:38.095242,garcinia_xanthochymus:38.095242):38.095242,garcinia_cowa:76.190483):38.095238,calophyllum_polyanthum:114.285721):38.095245,cratoxylum_cochinchinense:152.380966):38.095230):38.095245):28.571411,((((((elaeocarpus_glabripetalus.var.alatus:36.734692,elaeocarpus_sphaerocarpus:36.734692):36.734692,(elaeocarpus_petiolatus:36.734692,elaeocarpus_varunua:36.734692):36.734692):36.734695,elaeocarpus_prunifolioides:110.204079):36.734688,(elaeocarpus_balansae:73.469383,elaeocarpus_rugosus:73.469383):73.469383):36.734695,elaeocarpus_austroyunnanensis:183.673462):36.734695,sloanea_tomentosa:220.408157):36.734695):28.571442):19.047607,(((((((((citrus_japonica:43.537415,citrus_maxima:43.537415):43.537415,(glycosmis_esquirolii:43.537415,glycosmis_lucida:43.537415):43.537415):43.537415,acronychia_pedunculata:130.612244):43.537415,((((chukrasia_tabularis:43.537415,toona_ciliata:43.537415):43.537415,melia_azedarach:87.074829):43.537415,(((aglaia_elaeagnoide:36.281178,aglaia_perviridis:36.281178):36.281178,aglaia_lawii:72.562355):36.281181,(chisocheton_cumingianus:87.074829,(cipadessa_baccifera:65.306122,((dysoxylum_densiflorum:21.768707,dysoxylum_lenticellatum:21.768707):21.768707,(dysoxylum_gotadhora:21.768707,dysoxylum_sp:21.768707):21.768707):21.768707):21.768707):21.768707):21.768707):21.768707,(ailanthus_triphysa:76.190475,picrasma_javanica:76.190475):76.190475):21.768707):21.768707,(harpullia_cupanioides:156.734695,(sapindus_rarak:117.551018,(arytera_littoralis:78.367348,(nephelium_chryseum:39.183674,pometia_pinnata:39.183674):39.183674):39.183670):39.183678):39.183670):21.768707,(((garuga_floribunda.var.gamblei:54.421768,garuga_pinnata:54.421768):54.421768,(canarium_subulatum:54.421768,canarium_tonkinense:54.421768):54.421768):54.421768,((semecarpus_reticulata:54.421768,mangifera_sylvatica:54.421768):54.421768,toxicodendron_succedaneum:108.843536):54.421768):54.421768):21.768707,(aquilaria_yunnanensis:199.546478,((((pterospermum_lanceifolium:39.909298,pterospermum_menglunense:39.909298):39.909298,sterculia_lanceolata:79.818596):39.909294,kydia_glabrescens:119.727890):39.909302,(colona_floribunda:79.818596,microcos_chungii:79.818596):79.818596):39.909286):39.909302):21.768707,turpinia_pomifera:261.224487):21.768707,((((((((syzygium_forrestii:31.443687,syzygium_megacarpum:31.443687):31.443687,syzygium_cathayense:62.887375):31.443687,(syzygium_brachythyrsum:47.165531,syzygium_polypetaloideum:47.165531):47.165531):31.443687,(syzygium_balsameum:62.887375,syzygium_oblatum:62.887375):62.887375):31.443695,decaspermum_parviflorum:157.218445):31.443680,memecylon_caeruleum:188.662125):31.443695,(duabanga_grandiflora:110.052910,lagerstroemia_tomentosa:110.052910):110.052910):31.443680,terminalia_myriocarpa:251.549500):31.443695):21.768707):19.047607,((((((((((aidia_yunnanensis:29.437227,tarennoidea_wallichii:29.437227):29.437227,(wendlandia_scabra:29.437227,wendlandia_tinctoria.subsp.intermedia:29.437227):29.437227):29.437229,canthium_horridum:88.311684):29.437225,(saprosma_ternata:88.311684,(metadina_trichotoma:58.874454,(nauclea_officinalis:29.437227,neonauclea_tsaiana:29.437227):29.437227):29.437229):29.437225):29.437233,((alstonia_rostrata:49.062046,alstonia_scholaris:49.062046):49.062046,wrightia_pubescens:98.124092):49.062050):29.437225,(((((radermachera_microcalyx:29.437227,mayodendron_igneum:29.437227):29.437227,markhamia_stipulata:58.874454):29.437229,stereospermum_colais:88.311684):29.437225,(gmelina_arborea:78.499275,(callicarpa_arborea:39.249638,vitex_quinata.var.puberula:39.249638):39.249638):39.249634):29.437233,ehretia_sp:147.186142):29.437225):29.437225,(((apodytes_dimidiata:51.515148,apodytes_sp:51.515148):51.515148,pittosporopsis_kerrii:103.030296):51.515144,gomphandra_tetrandra:154.545441):51.515152):29.437225,((((schefflera_bodinieri:47.099564,schefflera_fengii:47.099564):47.099564,schefflera_octophylla:94.199127):47.099564,heteropanax_fragrans:141.298691):47.099564,(trevesia_palmata:125.598839,(macropanax_dispermus:62.799419,macropanax_undulatus:62.799419):62.799419):62.799416):47.099564):29.437241,(barringtonia_fusicarpa:231.818176,((((((camellia_sinensis.var.assamica:33.116882,schima_wallichii:33.116882):33.116882,(ternstroemia_gymnanthera:33.116882,eurya_groffii:33.116882):33.116882):33.116882,(symplocos_cochinchinensis:49.675323,symplocos_sp:49.675323):49.675323):33.116882,saurauia_cerea:132.467529):33.116882,styrax_tonkinensis:165.584412):33.116882,((diospyros_kaki.var.silvestris:105.974022,(ardisia_quinquegona:52.987011,ardisia_solanacea:52.987011):52.987011):52.987007,(((sarcosperma_arboreum:39.740257,sarcosperma_kachinense:39.740257):39.740257,sarcosperma_kachinense.var.simondii:79.480515):39.740265,pouteria_grandifolia:119.220779):39.740250):39.740265):33.116882):33.116882):29.437225,mastixia_pentandra.subsp.chinensis:294.372284):29.437225):19.047638,(meliosma_arnottiana:171.428574,meliosma_rigida:171.428574):171.428574):19.047607,(livistona_speciosa:180.952377,pandanus_tectorius:180.952377):180.952377):19.047638,((((((((((((litsea_glutinosa:29.304029,litsea_sp:29.304029):29.304029,(litsea_martabanica:29.304029,litsea_szemaois:29.304029):29.304029):29.304035,(litsea_monopetala:43.956047,litsea_salicifolia:43.956047):43.956047):29.304024,machilus_rufipes:117.216118):29.304039,alseodaphne_petiolaris:146.520157):29.304031,actinodaphne_henryi:175.824188):29.304016,alseodaphne_andersonii:205.128204):29.304031,(phoebe_puwenensis:156.288162,(phoebe_lanceolata:78.144081,phoebe_minutiflora:78.144081):78.144081):78.144073):29.304031,lindera_metcalfiana.var.dictyophylla:263.736267):29.304047,(cinnamomum_tenuipile:195.360214,(cinnamomum_bejolghota:97.680107,cinnamomum_pingbienense:97.680107):97.680107):97.680099):29.304016,(persea_tenuipilis:268.620270,((cryptocarya_acutifolia:107.448112,cryptocarya_yunnanensis:107.448112):107.448112,((beilschmiedia_robusta:80.586082,beilschmiedia_sp2:80.586082):80.586082,((beilschmiedia_fasciata:53.724056,beilschmiedia_purpurascens:53.724056):53.724056,(beilschmiedia_roxburghiana:53.724056,beilschmiedia_sp1:53.724056):53.724056):53.724052):53.724060):53.724045):53.724060):29.304047,((myristica_yunnanensis:241.130325,((horsfieldia_amygdalina:90.423874,knema_tenuinervia:90.423874):90.423874,(horsfieldia_kingii:120.565163,(horsfieldia_prainii:60.282581,knema_elegans:60.282581):60.282581):60.282585):60.282578):60.282578,((magnolia_henryi:125.588707,michelia_baillonii:125.588707):125.588707,((((miliusa_tenuistipitata:50.235481,pseuduvaria_indochinensis:50.235481):50.235481,mezzettiopsis_creaghii:100.470963):50.235489,polyalthia_simiarum:150.706451):50.235474,mitrephora_maingayi:200.941925):50.235489):50.235489):50.235474):29.304016):19.047619)euphyllophyte:1.000000;
